# 

**Published:** 1887-09

**Authors:** 


### BOOKS AND PAMPHLETS RECEIVED.

On the Laws of Blockade. A Thesis for the degree of Civil Law presented to McGill University, Montreal. By Thomas Nichol, B. C. L., February 9th, 1887. The writer of the above thesis is a homœopathic physician. He received his degree April 9th, and thus adds the title D. C. L. to his other title M. D. We are not competent to criticise the above pamphlet.

Researches into the Etiology of Dengue. By J. W. McLaughlin, M. D.

Notes on Consumption and its Treatment. By Dr. Stammers Morrisson.

Novel Methods of Treating Diseases of the Middle Ear. By Seth L. Bishop, M. D.

Introduction to the Study of the Homœopathic Materia Medica. A lecture by C. L. Cleveland, A. M., M. D., Professor of Materia Medica.

Archives of Gynæcology for July.

Homœopathic League Tract No. 14. "The Royal College of Physicians of London and Homœopathy."

First Annual Report of the Homœopathic League for the year ending April 30th, 1887.

Fort Wayne Journal of the Medical Sciences, July.

A TREATISE ON SOME DISEASES OF THE BONES AND LIGAMENTS OF THE SPINE. S. M. Cate, M. D.

PERSISTENT PAIN AFTER ABDOMINAL SECTION. By James B. Hunter, M. D.

ANNUAL ADDRESS READ BEFORE THE HOMŒOPATHIC MEDICAL SOCIETY OF OHIO. By Albert Claypool, M. D.

HOW TO SUCCEED AS PHYSICIANS. An address before the Rhode Island Homœopathic Medical Society. By James B. Bell, M. D.

MEDICAL CLASSICS, Vol. I, No. 1.

LITHÆMIA. By J. W. Dowling, M. D.

INAUGURAL ADDRESS UPON THE WORK OF THE NEW YORK SOCIETY FOR MEDICO-SCIENTIFIC INVESTIGATION. By Walter Y. Cowl, M. D.

OMIOPATIA HAHNEMANNIANA E OMIOPATIA METICCIA DEL DOTT, ATTILIO MATTOLI. FOLLICULAR AMYGDALITIS. A. Jacobi, M. D.

We also desire to acknowledge the receipt of announcements and catalogues of the following colleges: New York Homœopathic Medical College and Hospital, University of City of New York; College of Physicians and Surgeons, Baltimore, M. D.; Chicago Homœopathic Medical College; Bellevue Hospital and Medical College; College of Physicians and Surgeons in the City of New York, being the medical department of Columbia College; Homœopathic Medical College of Missouri; Long Island College Hospital, Brooklyn.

NOTICE.

*All articles for publication, books for review, business communications, etc., must be sent to Editors, 1123 Spruce Street, Philadelphia.*

*Subscribers will please notice that this Journal will be sent until arrears are paid and it is ordered discontinued.*

PHILADELPHIA:

No. 1123 SPRUCE STREET.

NEW YORK.
A. L. CHATTERTON & COMPANY, PUBLISHER'S AGENTS.

LONDON:
ALFRED HEATH & CO., SOLE AGENTS FOR GREAT BRITAIN,
114 Ebury Street, Eaton Square.

*Yearly Subscription, $2.50, invariably in advance. Single numbers, 25 cts.*

Entered at the Philadelphia Post-Office as Second-Class Mail Matter.

**All subscriptions are due in advance; subscribers will therefore please forward their subscriptions *at once;* let *each* subscriber see that *his* account is settled promptly.**

***Subscribers are respectfully requested to make all checks and money-orders payable to THE HOMŒOPATHIC PHYSICIAN, and not to either of the Editors.***

